# Supplementary material for: More than ticking boxes: Training Lyme disease education ambassadors to meet outreach and surveillance challenges in Québec, Canada
Source: PLoS One. 2021 Oct 12;16(10):e0258466. doi: 10.1371/journal.pone.0258466 (PMC8509862; doi:10.1371/journal.pone.0258466)

S1 Table

QUESTIONNAIRE (ORIGINAL, FRENCH)

ÉVALUATION DE L'ATELIER DE FORMATION DES AMBASSADEURS AU SUJET DES TIQUES ET DE LA MALADIE DE LYME (INSPQ)

*Rempli par les ambassadeurs et les invités après avoir assisté à un atelier de formation
*Réponses anonymes, questionnaire en ligne sur la plateforme SurveyMonkey®

Q1– EN UTILISANT L'ÉCHELLE SUIVANTE, VEUILLEZ INDIQUER À QUEL POINT CETTE ACTIVITÉ DE FORMATION VOUS A PERMIS DE RÉPONDRE À CHACUN DES OBJECTIFS D'APPRENTISSAGE ?

CHOIX DE RÉPONSES : PAS DU TOUT (0pt) / PEU (1pt) / ASSEZ (2pts) / BEAUCOUP (3pts) / ENTIÈREMENT (4pts) / N/A

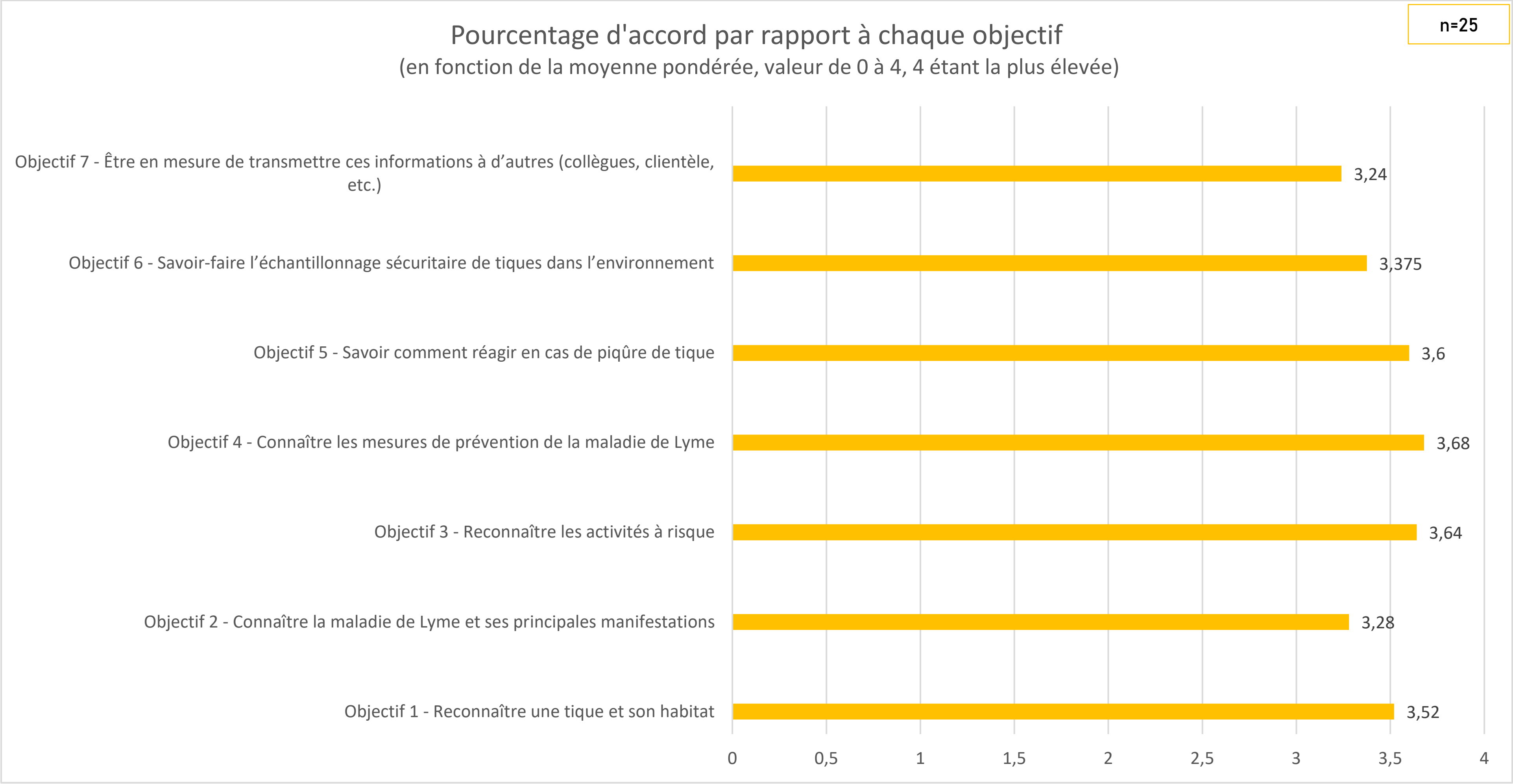

Q2. EN UTILISANT L'ÉCHELLE SUIVANTE, INDIQUEZ VOTRE NIVEAU D'ACCORD QUANT À CHACUN DES ÉNONCÉS CI-DESSOUS:

CHOIX DE RÉPONSES : TOUT À FAIT EN DÉSACCORD (0pt) / PLUTÔT EN DÉSACCORD (1pt) / PLUTÔT NEUTRE (2pts) / PLUTÔT D'ACCORD (3pts) / TOUT À FAIT D'ACCORD (4pts) / N/A

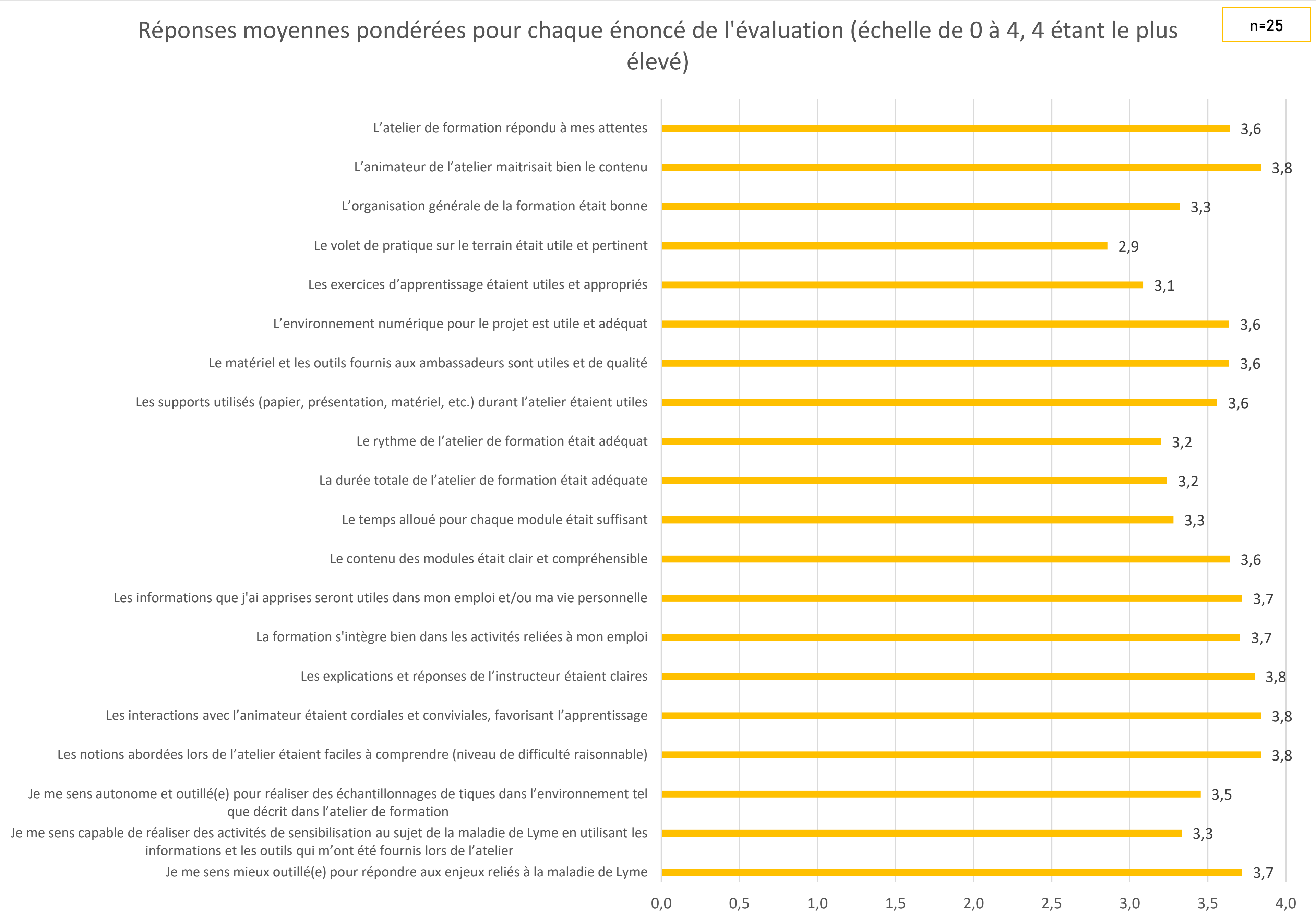

QUESTIONNAIRE (TRANSLATED, ENGLISH)
EVALUATION OF THE AMBASSADOR TRAINING WORKSHOP (INSPQ)

*Completed by ambassadors and guests after attending a training workshop
*Anonymous responses, online questionnaire on SurveyMonkey® platform

Q1– USING THE FOLLOWING SCALE, PLEASE INDICATE HOW WELL THIS TRAINING ACTIVITY HELPED YOU MEET EACH OF THE LEARNING OBJECTIVES. ANSWER OPTIONS : NOT AT ALL (0 pt) / A LITTLE (1pt) / ENOUGH (2pts) / A LOT (3pts) / COMPLETELY (4pts) / N/A

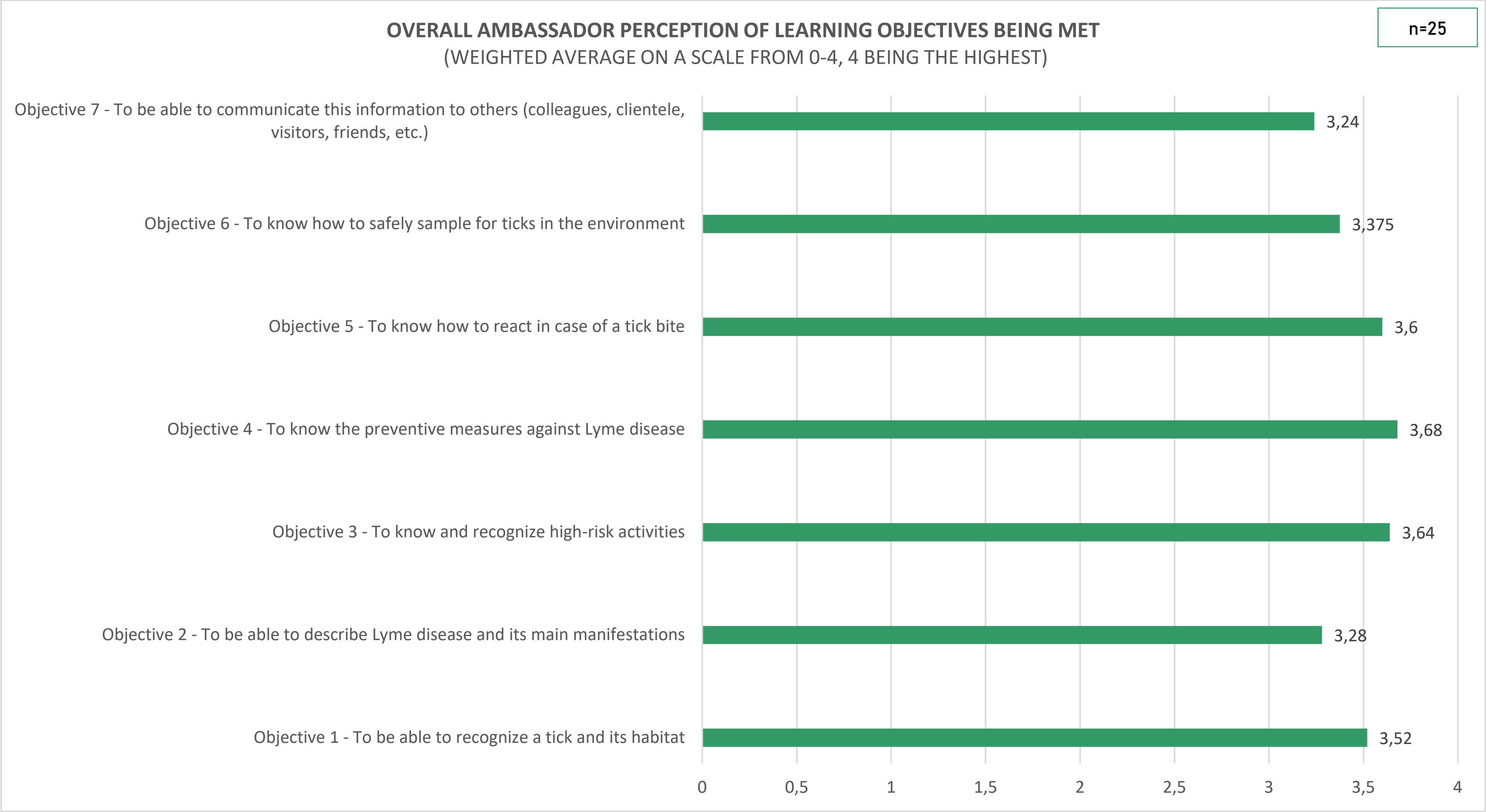

Q2. USING THE FOLLOWING SCALE, INDICATE YOUR LEVEL OF AGREEMENT WITH EACH OF THE STATEMENTS BELOW

ANSWER OPTIONS : STRONGLY DISAGREE (0 pt) / SOMEWHAT DISAGREE (1pt) / RATHER NEUTRAL (2pts) / MOSTLY AGREE (3pts) / TOTALLY AGREE (4pts) / N/A

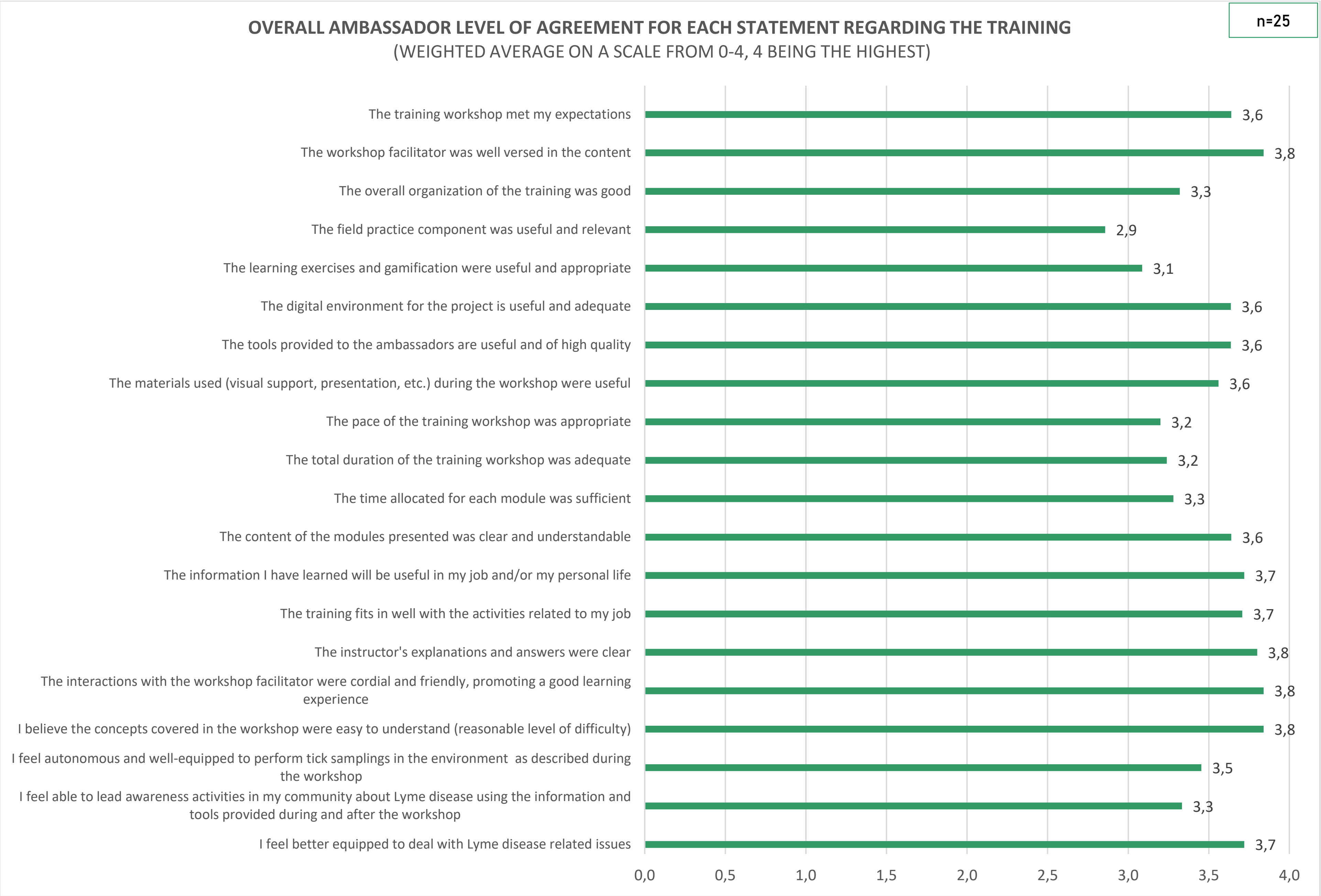

Supplement: S1 Table — (PDF) [file pone.0258466.s001.pdf]
