# Supplementary material for: More than ticking boxes: Training Lyme disease education ambassadors to meet outreach and surveillance challenges in Québec, Canada
Source: PLoS One. 2021 Oct 12;16(10):e0258466. doi: 10.1371/journal.pone.0258466 (PMC8509862; doi:10.1371/journal.pone.0258466)
Supplement: S2 Table — (PDF) [file pone.0258466.s002.pdf]

S2 Table. List of ambassador-led activities (sampling and outreach)

OUTREACH ACTIVITIES	MUNICIPALITY	REGION	NB OF PARTICIPANTS	SMALL DESCRIPTION OF THE ACTIVITY
	Québec	R03 - Capitale Nationale	Open to all (wide public)	Open information sharing, use of awareness-raising tools
	Bristol, Clarendon, Aylmer (Gatineau)	R07 - Outaouais	79	Shared information with the "Volunteers for Conservation" groups during outings.
	Bristol, Gatineau, Luskville, etc.	R07 - Outaouais	32	Open information sharing, use of awareness-raising tools within their organization
	Multiple locations	R06 - Montréal	27	Sharing of electronic outreach information and tools with employees and interns
	Lévis	R12 - Chaudière-Appalaches	11	Occupational health: shared outreac information and tools with colleagues
	Drummondville	R04 - Mauricie-Centre-du-Québec	5	Occupational health: shared outreac information and tools with colleagues
	Drummondville	R04 - Mauricie-Centre-du-Québec	30	Evening talk on ticks and LD with their clientele
	Nicolet/Yamaska, Drummond, Artabas	R04 - Mauricie-Centre-du-Québec	30	5 training/awareness-raising activities for at-risk job categories and municipal workers (1 per administrative region)
	Sutton	R16 - Montérégie	230	Awareness-raising activities with students of the "Jeunes-Nature" Program during school outings
	Sutton	R16 - Montérégie	300	Summer camp : Awareness-raising activities with parents and campers (spending 1 week in the forest)
	St-Jean-sur-le-Richelieu	R16 - Montérégie	7	Outreach information on LD and ticks added to another training planned
	Mont-Saint-Grégoire	R16 - Montérégie	350	Summer camp : Awareness-raising activities added to regular programmation
	Mont-Saint-Grégoire	R16 - Montérégie	10	Outreach information provided while hiking on the mountain
	Granby	R05 - Estrie	307	Prevention and outreach booth on Lyme disease: open to all information
	Plaisance	R07 - Outaouais	130	Outreach and information sharing at the end of talks (part of regular schedule)
	Bromont	R16 - Montérégie	8	"Community researchers" Workshop
	Bromont	R16 - Montérégie	25	Conference for the general public about LD and ticks
	Bromont	R16 - Montérégie	37	Tick identification workshop
	Bromont	R16 - Montérégie	17	Focus group - What are the citizens' overall perception on LD + outreach
	Bromont	R16 - Montérégie	56	General public information session
	Bromont	R16 - Montérégie	7	"Community researchers" Workshop
	Bromont	R16 - Montérégie	37	Tick identification workshop
	Beloeil	R16 - Montérégie	15	Outreach information on LD and ticks provided to field/outdoor workers in their organizatic
	Beloeil	R16 - Montérégie	30	Prevention and outreach booth on Lyme disease: open to all information
	Lévis	R12 - Chaudière-Appalaches	30	Open information sharing with staff members + use of awareness-raising tools in local municipality offices
	Notre-Dame-de-Bonsecours / Monteb	R07 - Outaouais	Open to all (wide public)	Sharing of awareness materials (brochures, posters)
	Gatineau, Hull	R07 - Outaouais	18	Presentation with local community of ortnithologists: "Lyme Disease: Birders at risk"
TOTAL	15+	7 regions	1828	

SAMPLING ACTIVITIES	MUNICIPALITY	REGION	NB OF TICK(S)	DISTANCE SAMPLED (meters)
	N/A	R03 - Capitale Nationale	0	2000
	N/A	R03 - Capitale Nationale	0	2000
	N/A	R04 - Mauricie-Centre-du-Québec	5	2000
	N/A	R05 - Estrie	0	2000
	N/A	R07 - Outaouais	0	2000
	N/A	R12 - Chaudière-Appalaches	0	2000
	N/A	R12 - Chaudière-Appalaches	0	2000
	N/A	R12 - Chaudière-Appalaches	1	2000
	N/A	R12 - Chaudière-Appalaches	0	2000
	N/A	R12 - Chaudière-Appalaches	0	2000
	N/A	R12 - Chaudière-Appalaches	0	2000
	N/A	R12 - Chaudière-Appalaches	0	2000
	N/A	R12 - Chaudière-Appalaches	0	2000
	N/A	R07 - Outaouais	0	2000
	N/A	R07 - Outaouais	0	2000
	N/A	R07 - Outaouais	0	2000
	N/A	R07 - Outaouais	0	2000
	N/A	R07 - Outaouais	3	2000
	N/A	R07 - Outaouais	2	2000
	N/A	R07 - Outaouais	0	2000
	N/A	R07 - Outaouais	0	2000
	N/A	R07 - Outaouais	0	2000
	N/A	R06 - Montréal	0	2000
	N/A	R16 - Montérégie	0	2000
	N/A	R16 - Montérégie	0	2000
	N/A	R16 - Montérégie	0	2000
	N/A	R16 - Montérégie	0	2000
	N/A	R16 - Montérégie	0	2000
TOTAL	13+	7 regions	11 ticks	55 km

NOTE: Exact ocation information is confidential.
