# Supplementary material for: More than ticking boxes: Training Lyme disease education ambassadors to meet outreach and surveillance challenges in Québec, Canada
Source: PLoS One. 2021 Oct 12;16(10):e0258466. doi: 10.1371/journal.pone.0258466 (PMC8509862; doi:10.1371/journal.pone.0258466)
Supplement: S3 Table — (PDF) [file pone.0258466.s003.pdf]

S3 Table. Pathogen screening in ticks sampled by ambassadors

DRAG SAMPLER	SUBMITTER	STAGE	SPECIES	NML No.	DATE EXTRACTED	REGION (RSS)	*B. burgdorferi*			*B. miyamotoi*			*A. phagocytophilum*			*Babesia mic*		Powassan Encephalitis virus				
							23S	ospA	Status	flaB	Statu	msp2	msp2	Statu	18S	cctn	Status	POW NS5	POW	Statu	DTV NS5	Status
AMBASSADOR 2019	INSPQ	Nymph	*Ixodes sca*	AS19-891	2019-12-02	Maurici	40		Negative		Nega	40		Nega	40		Negat	40		Negative		Negative
AMBASSADOR 2019	INSPQ	Nymph	*Ixodes sca*	AS19-892	2019-12-02	Outaoua	40		Negative		Nega	40		Nega	40		Negat	40		Negative		Negative
AMBASSADOR 2019	INSPQ	Nymph	*Ixodes sca*	AS19-893	2019-12-02	Outaoua	40		Negative		Nega	40		Nega	40		Negat	40		Negative		Negative
AMBASSADOR 2019	INSPQ	Nymph	*Ixodes sca*	AS19-894	2019-12-02	Outaoua	32,2	34,4	Positive	40	Nega	40		Nega	40		Negat	40		Negative		Negative
AMBASSADOR 2019	INSPQ	Nymph	*Ixodes sca*	AS19-895	2019-12-02	Maurici	40		Negative		Nega	40		Nega	40		Negat	40		Negative		Negative
AMBASSADOR 2019	INSPQ	Male	*Ixodes sca*	AS19-896	2019-12-02	Chaudiè	40		Negative		Nega	40		Nega	40		Negat	40		Negative		Negative
AMBASSADOR 2019	INSPQ	Nymph	*Ixodes sca*	AS19-897	2019-12-02	Maurici	40		Negative		Nega	40		Nega	40		Negat	40		Negative		Negative
AMBASSADOR 2019	INSPQ	Nymph	*Ixodes sca*	AS19-898	2019-12-02	Maurici	40		Negative		Nega	40		Nega	40		Negat	40		Negative		Negative
AMBASSADOR 2019	INSPQ	Nymph	*Ixodes sca*	AS19-899	2019-12-02	Maurici	38,3	38,1	Positive	40	Nega	40		Nega	40		Negat	40		Negative		Negative
AMBASSADOR 2019	INSPQ	Female	*Ixodes sca*	AS19-900	2019-12-02	Outaoua	40		Negative		Nega	40		Nega	40		Negat	40		Negative		Negative
AMBASSADOR 2019	INSPQ	Male	*Ixodes sca*	AS19-901	2019-12-02	Outaoua	40		Negative		Nega	40		Nega	40		Negat	40		Negative		Negative
TOTAL		8 Nymph, 2 males, 1 female							2 positives		None			None			None			None		None

NOTE: Locations and sampler identities are confidential.
